# Harnessing artificial intelligence of things for cardiac sensing: current advances and network-based perspectives

**DOI:** 10.3389/fpubh.2025.1569887

**Published:** 2025-07-16

**Authors:** Hao Ren, Fengshi Jing, Yongcong Ma, Ruining Wang, Chaocheng He, Yufan Wang, Jiandong Zhou, Yu Sun

**Affiliations:** ^1^Institute for Healthcare Artificial Intelligence Application, The Affiliated Guangdong Second Provincial General Hospital of Jinan University, Guangzhou, China; ^2^Guangzhou Key Laboratory of Smart Home Ward and Health Sensing, Guangzhou, China; ^3^Faculty of Data Science, City University of Macau, Taipa, Macao SAR, China; ^4^School of Information Management, Wuhan University, Wuhan, China; ^5^Department of Industrial Engineering and Management, Shanghai Jiao Tong University, Shanghai, China; ^6^Department of Family Medicine and Primary Care, LKS Faculty of Medicine, The University of Hong Kong, Hong Kong, Hong Kong SAR, China; ^7^Department of Cardiac Intensive Care Unit, The Affiliated Guangdong Second Provincial General Hospital of Jinan University, Guangzhou, China

**Keywords:** artificial intelligence of things (AIoT), cardiac sensing, edge computing, deep learning techniques, precision medicine, scientometrics, data privacy protection, large language models

## Abstract

**Background:**

With the rapid advancements in science and technology, artificial intelligence (AI) has become increasingly integral to various medical applications, including medical devices and assistive healthcare tools. Extensive research highlights the significant potential of AI in the development of Internet of Things (IoT)-enabled medical devices, particularly in the field of cardiac sensing.

**Methods:**

This study explores and synthesizes current advancements and future directions of AI-driven IoT applications in cardiac sensing, highlighting their significance. Utilizing a bibliometric approach, we visualize key focus areas, emerging trends, and the evolutionary trajectory of this interdisciplinary field.

**Results:**

As of December 2024, relevant literature at the intersection of IoT, cardiac sensors, and AI was systematically retrieved from the SCIE and ESCI indices. Using CiteSpace, we conducted a comprehensive visualization analysis of countries/regions, academic publications, organizations, authors, citations, and key terminologies. A total of 2,128 papers were included in the analysis.

**Conclusion:**

From our perspective, current advancements in AI-powered IoT cardiac sensors primarily focus on optimizing AI algorithms, such as deep learning techniques, and enhancing the functionality of smart wearable devices for precision medicine. Looking ahead, we anticipate that this field will increasingly prioritize data privacy protection, particularly in the era of large language models, to address emerging challenges and ensure sustainable growth. In summary, we need to continue harnessing the power of AI-powered IoT for cardiac sensing as part of public health strategies to enable early detection of heart diseases.

## 1 Introduction

Cardiovascular diseases (CVDs) remain the foremost cause of mortality worldwide, accounting for an estimated 17.9 million deaths in 2019, or 32% of all global fatalities (1). According to the Global Burden of Disease Study 2019, ischemic heart disease and stroke together contributed the largest share of disability-adjusted life years (DALYs) among over 369 conditions analyzed across 204 countries and territories (2). These figures underscore the critical need for early detection and continuous monitoring strategies to reduce both acute events and long-term complications.

Traditional cardiac monitoring modalities—such as standard 12-lead electrocardiography (ECG) and 24-h Holter recording—are well established in clinical practice but present significant drawbacks. Conventional ECG systems typically involve bulky hardware and multiple wired electrodes, which can cause discomfort and limit patient mobility (3). Moreover, assessments are often episodic rather than continuous, potentially missing transient arrhythmias or subtle physiological changes; efforts to develop wireless systems have led to platforms that reduce cables but still face challenges in ensuring signal fidelity and patient compliance (4).

Artificial intelligence (AI) refers to the simulation of human cognitive functions by machines, particularly computer systems capable of perceiving their environment, learning from data, and making decisions to perform tasks that traditionally require human intelligence (5). The Internet of Things (IoT) is a worldwide network of uniquely addressable physical objects—such as sensors, actuators, and embedded systems—that interact and exchange data over standard communication protocols (6). The convergence of these two domains gives rise to the Artificial Intelligence of Things (AIoT), wherein AI algorithms are embedded within IoT infrastructures to enable on-device data analytics, real-time decision-making, and adaptive system behaviors across cloud, fog, and edge environments (7). In healthcare applications, cardiac sensing denotes the non-invasive acquisition of physiological signals related to heart activity, including electrical potentials via electrocardiography, volumetric pulse changes via photoplethysmography, and mechanical vibrations via mechanocardiography, to monitor cardiac function and detect anomalies continuously (8).

The advent of the AIoT—the convergence of IoT-enabled sensors with on-device and cloud-based AI—offers a paradigm shift for cardiac sensing. AIoT systems enable real-time data acquisition, edge-based inference, and seamless model updates, thus minimizing latency and preserving data privacy (9). For example, Edge2Analysis demonstrates a lightweight atrial fibrillation detection platform that runs on resource-constrained embedded processors, achieving diagnostic accuracy comparable to cloud-based solutions while reducing communication overhead (10). Convolutional neural networks (CNNs) have become integral to automated ECG analysis, enabling end-to-end feature extraction from raw waveform data; in a landmark study, Hannun et al. trained a 34-layer CNN on over 90,000 single-lead ECG recordings to classify 12 rhythm types, achieving an average ROC AUC of 0.97—performance on par with expert cardiologists (11). In parallel, long short-term memory (LSTM) networks excel at modeling the temporal dynamics of sequential ECG signals; Yildirim (12) proposed a bidirectional LSTM framework with wavelet-based preprocessing that decomposed ECG signals into multiscale sequences, attaining 99.39% classification accuracy across five heartbeat categories on the MIT-BIH Arrhythmia Database (12). These examples underscore the complementary strengths of CNN and LSTM architectures for robust arrhythmia detection in AIoT-enabled cardiac sensing applications. In addition to continuous sensing and arrhythmia detection, AIoT-enabled cardiac platforms are increasingly applied to remote patient monitoring and home-based rehabilitation. For instance, implantable wireless sensors such as the CardioMEMS^™^ HF System allow clinicians to track pulmonary artery pressures in heart failure patients and intervene proactively, resulting in a 37% reduction in HF hospitalizations over 15 months (13). Likewise, smartphone-based cardiac rehabilitation programs integrate wearable activity trackers, on-device guidance, and cloud-mediated telecoaching to deliver exercise prescriptions and education remotely; in a randomized trial, a mobile home-based CR intervention achieved an 85% completion rate and similar gains in peak VO₂ compared with center-based programs (14). Practical deployment of AIoT cardiac sensing is challenged by high false-positive rates and model overfitting. Dang et al. (15) reported that wearable cardioverter-defibrillators generated an average of 38.7 false alarms per patient over 71.5 days, mostly due to motion artefacts, eroding patient trust and adherence (15). Ensuring informed consent and transparency in AI decision-making—by clearly disclosing AI involvement and providing explainable rationales—is critical for upholding patient autonomy and trust in clinical deployments (16).

Despite these technological strides, the literature on AIoT for cardiac monitoring remains fragmented, with most studies addressing individual components—such as sensor design, algorithmic performance, or communication protocols—without offering an integrated field-wide perspective (17). Bibliometric and scientometric methods provide robust frameworks for mapping research landscapes, uncovering co-authorship networks, and tracking the evolution of key topics over time. Global patterns of AI research collaboration have been extensively characterized through bibliometrics, revealing dominant countries, institutions, and thematic hotspots (18). Tools like CiteSpace facilitate the visualization of research fronts and intellectual bases, detecting citation bursts and pivotal works within a domain (19).

Despite significant advancements in AIoT for cardiac sensing, the existing research landscape remains fragmented, often addressing specific technological aspects such as sensor development, algorithmic refinement, or communication standards individually rather than providing a cohesive, integrative perspective. To bridge this gap, our study employs a comprehensive bibliometric approach to systematically map the research published from 2018 to 2024 within the Web of Science Core Collection. Utilizing network analyses—including co-authorship, co-citation, and keyword co-occurrence—we elucidate patterns of scholarly collaboration, pinpoint critical technological advancements, and identify emerging research trends and persistent challenges. Additionally, we evaluate key publicly available datasets and highlight the pivotal role of machine learning techniques—ranging from classical algorithms like logistic regression and random forests to advanced deep learning architectures such as CNNs and LSTMs—in transforming cardiac sensing data into actionable clinical insights. By emphasizing the synergy between deep learning methodologies, precision medicine applications, robust data privacy protocols, and efficient algorithm deployment in edge computing environments, particularly crucial in the context of evolving large language models (LLMs), this research provides an authoritative, integrated overview. Ultimately, our objective is to guide future innovation and foster interdisciplinary collaboration, thus enhancing the translational potential of AIoT-enabled cardiac sensing in clinical practice and public health strategies.

## 2 Materials and methods

In terms of data, we began by collecting recent literature from the Web of Science (WoS). The WoS Core Collection, provided by Clarivate Analytics, is a multidisciplinary citation database containing over 21,000 peer-reviewed journals, 300,000 conference proceedings, and 134,000 books, covering 254 subject areas. The Science Citation Index Expanded (SCIE), a core index of WoS, includes 9,500 globally recognized journals and 61 million publications across 182 natural science categories. The Emerging Sources Citation Index (ESCI) comprises 8,000 high-quality journals and 4 million publications across 254 categories. For this study, we primarily used the SCIE and ESCI indices as data sources. Next, we focused on data from January 1, 2018, to December 26, 2024, excluding subsequent updates to ensure consistency. For the topic “AI of Things for Heart Sensing,” our search query was: TS = (((“Artificial Intelligence” OR AI OR “Machine Learning” OR “Deep Learning” OR CNN OR LSTM OR “Neural Network” OR “Data Mining”) AND (“Heart” OR Cardiac OR Cardiovascular OR Atrial OR Ventricular OR Arrhythmia OR “Heart Disease” OR “Cardiology”) AND (“IoT” OR “Wearable Devices” OR “Sensors” OR “Smart Devices” OR “ECG” OR “Medical Monitoring”))) AND PY = (2018–2024).

**Figure 1:**
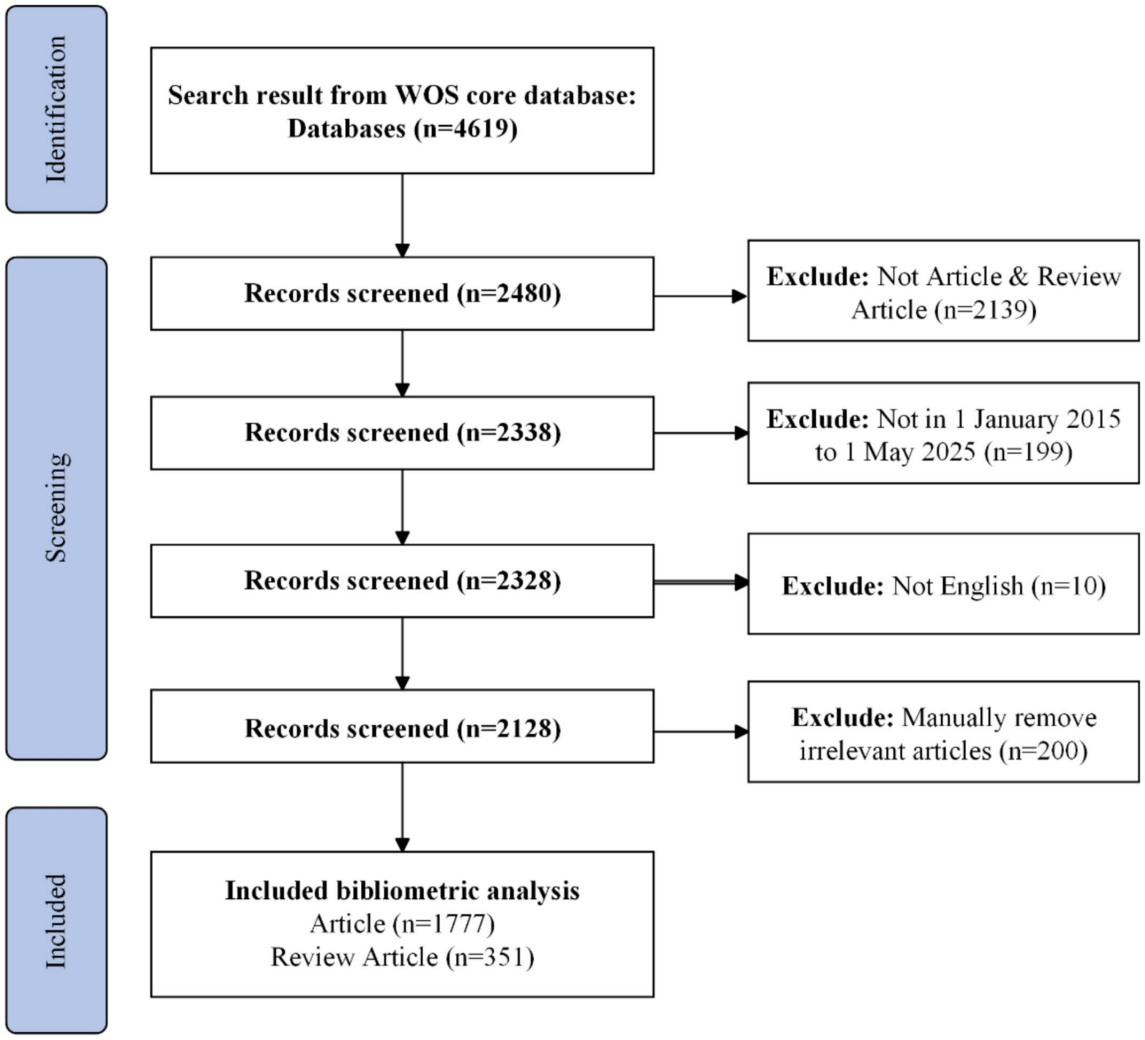
Flowchart of the search strategy in the study.

Prior to 2018, AIoT-enabled cardiac monitoring was largely confined to proof-of-concept studies and small-scale pilots; from 2018 onward, however, the field has witnessed a pronounced shift toward large-scale clinical and consumer implementations (20–22). Figure 1 depicts our stepwise bibliometric workflow for mapping the AIoT cardiac-sensing literature. We began with a topic-based search of the Web of Science Core Collection—covering both the SCIE and the ESCI—and restricted our dataset to publications dated January 1, 2018 through December 26, 2024. All retrieved records were exported in plain-text format with full bibliographic details and cited references, then imported into CiteSpace for successive analyses of co-authorship, co-citation, and keyword co-occurrence networks. This flowchart ensures a transparent and reproducible approach to data acquisition, preprocessing, and visualization in the present study.

Following this, we used the Web of Science search tool to perform a basic analysis of the number of publications, citation counts, and global trends. The records were then exported in “plain text format” as “full record and cited references” for further analysis in CiteSpace, a widely used software in many studies (23, 24).

CiteSpace is a bibliometric tool designed to reveal research trends and knowledge structures. It can perform citation analysis to identify key publications, research hotspots, and influential authors while supporting various data sources. CiteSpace also generates time-based visual maps to illustrate the evolution of a field. Its powerful visualization capabilities allow for intuitive exploration of citation links, co-citation, and bibliometric coupling, while its text mining features analyze keyword co-occurrence to highlight key topics and emerging trends. Therefore, it is a valuable tool for discussing current advances and future directions in this perspective article. Furthermore, we searched Scopus and PubMed; after limiting the results to SCI/SSCI publications, we confirmed that all of these were already captured by our Web of Science search.

## 3 Recent and current advances

### 3.1 Global trends of publications

**Figure 2:**
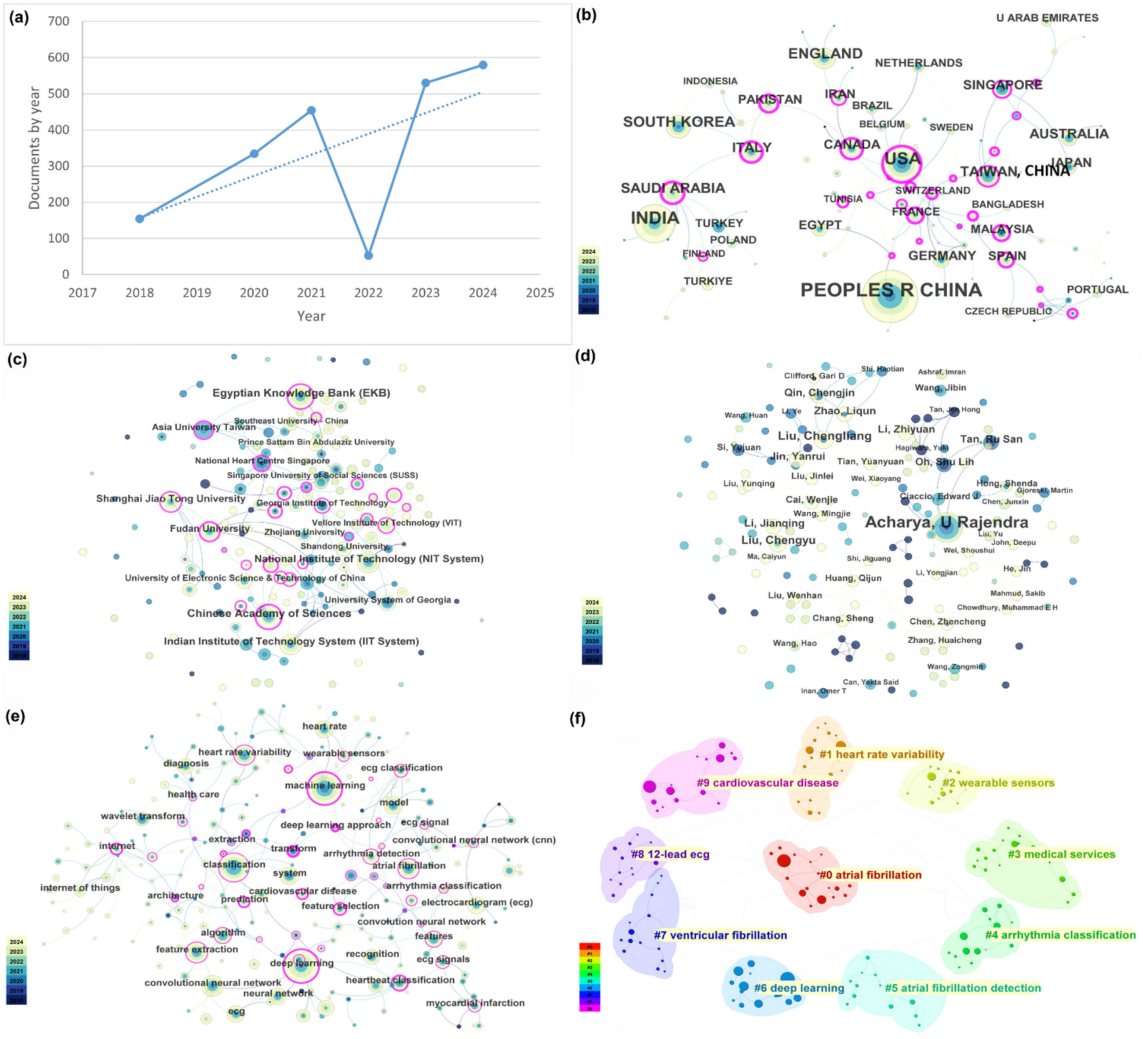
Network analysis of this research field. **(a)** Global trends of publications. **(b)** Global research productivity and influence network visualization map of countries and regions. **(c)** Network visualization of institutions. **(d)** Network visualization of authors. **(e)** Keyword co-occurrence visualization map. **(f)** Keyword clustering visualization map.

Over the past seven years (January 2018 to December 2024), a total of 2,128 publications on *AI of Things for Cardiac Sensing* were identified. As illustrated in Figure 2a, annual output in AI-driven IoT-cardiology research remained modest from 2018 to 2020, with fewer than 100 papers published each year. A slight dip occurred in 2022 (approximately 80 papers), potentially attributable to the global COVID-19 pandemic, which redirected medical research priorities toward pandemic-related studies. Despite this temporary decline, the field exhibited sustained growth overall, with a notable acceleration post-2019. Specifically, publication volumes surged by over 300% between 2020 and 2024, reflecting intensified research activity and technological maturation in AIoT-cardiac integration.

### 3.2 Network analysis of countries or regions

In the search for AIoT applications in heart sensors, publication data by country/region shows that the United States leads in the number of papers published, followed by China and India. These countries/regions also exhibit the strongest international collaboration networks (Figure 2b). While China’s publication output and collaboration ratio are generally lower, the United States is highly proactive in collaborating with other countries/regions. Overall, international collaboration is predominantly concentrated in developed countries/regions, while developing countries/regions lag behind in both publication output and cooperation.

### 3.3 Network analysis of institutions

According to Web of Science (WoS) statistics, more than 500 institutions have published research papers on the application of AIoT in heart sensors. Among them, the institutions with the highest publication counts are the Chinese Academy of Sciences and the Egyptian Knowledge Bank. Figure 2c illustrates the collaboration network among these institutions, where thicker lines represent stronger collaborative relationships. As shown in the figure, institutional collaboration is notably close, especially within domestic institutions. However, similar to the analysis of country or regional collaboration, there are still certain limitations in inter-institutional cooperation. For instance, while institutions within China have relatively strong collaboration ties, they exhibit insufficient depth of cooperation with institutions from other countries or regions. This phenomenon may be related to factors such as research resources, technological advantages, and the willingness to collaborate across different countries or regions, warranting further investigation.

### 3.4 Network analysis of authors

WOS records indicate that a total of 153 authors have published papers in the field of AIoT and heart sensors. Among these authors, the top five in terms of publication volume are Acharya U Rajendra (41 publications), Liu Chengliang (14 publications), Liu Chengyu (12 publications), Qin Chengjin (9 publications), and Jin Yanrui (9 publications). Specifically, Professor U Rajendra Acharya stands out as the most prolific scholar in this field. Since 2018, Professor Acharya has published 41 papers, establishing himself as a leading figure in this domain. His research focuses on the application of AIoT technologies in heart monitoring, particularly in data processing and algorithm optimization. His contributions have had a profound impact on the advancement of this field.

It is noteworthy that, among the top 20 authors, only five are from industry, with the remaining authors primarily from academia. This trend suggests that, while the industrial sector has played a crucial role in driving technological applications and commercialization, academia still holds a dominant position in this area of research. Academia has not only made significant contributions to fundamental research and technological innovation but also provided theoretical support for the deeper exploration of AIoT in heart sensors. Furthermore, academia’s leading role has fostered more interdisciplinary collaboration, particularly in fields such as computer science, biomedical engineering, and bioinformatics, providing strong support for the optimization of AIoT systems.

The collaboration relationships among authors are shown in Figure 2d. In this field, cooperation tends to be organized by country, with limited international collaboration. However, as international exchange and cooperation gradually increase, it is expected that this trend will improve in the future. Cross-border collaboration can promote the rapid dissemination and sharing of technologies, accelerating the application and innovation of AIoT technologies in the field of heart sensors.

Overall, the dominant position of academia, coupled with the contributions from industry, has mutually reinforced and driven the development of the IoT and heart sensor field. In the future, as international cooperation strengthens and interdisciplinary research deepens, the application prospects of AIoT in heart health monitoring will become even more promising.

### 3.5 Network analysis of keywords

Figure 2e shows a dense co-occurrence network in which “machine learning,” “deep learning,” “neural network” and “convolutional neural network” occupy the highest-degree nodes, each tightly connected to biomedical-signal terms (“ECG” “heart rate variability,” “arrhythmia detection”) and bridged by “wearable sensor” and “Internet of Things,” underlining the fusion of AI methods with IoT platforms for real-time cardiac monitoring. Figure 2f partitions these keywords into ten modular clusters—of which the most prominent are atrial fibrillation detection (#0), heart rate variability analysis (#1), wearable-sensor development (#2), deep learning architectures (#6) and broader cardiovascular disease applications (#9)—thereby mapping the principal thematic areas driving AIoT research in heart sensing.

### 3.6 Network analysis of citations

**Figure 3:**
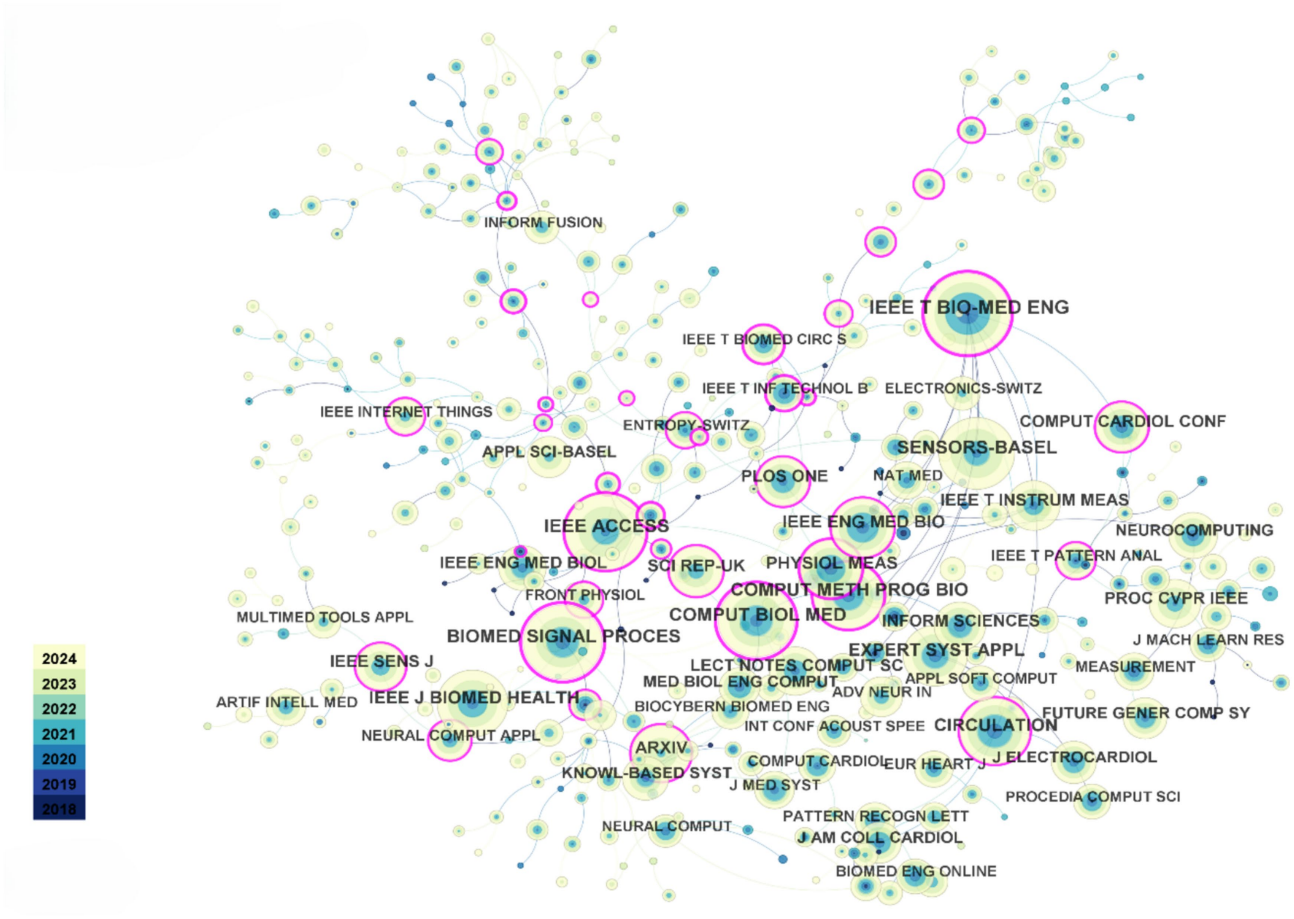
Journal co-citation relationship visualization. Each node represents a journal and is color-coded by modularity class.

Since 2018, the top five most-cited journals in the dataset are: IEEE Transactions on Biomedical Engineering (1,206 citations), IEEE Access (1,024 citations), Biomedical Signal Processing and Control (1,022 citations), Computers in Biology and Medicine (1,011 citations), and Sensors (970 citations). As shown in Figure 3, the co-citation network among these journals indicates strong and close connections, with their collaboration being globally widespread. Compared to the connections between countries and authors, the collaboration network among journals exhibits a more international and interdisciplinary character. This phenomenon suggests that research in the field of heart sensors has attracted significant global attention, and journal collaboration has played a key role in disseminating and sharing research outcomes in this area.

Regarding authors, since 2018, Goldberger AL has been cited 510 times, ranking first, followed by Acharya UR with 492 citations. These data reflect the significant positions and influence of these two scholars in the field, particularly in the application of AIoT technologies to heart sensors. Their work has laid a solid theoretical foundation and provided technical support for subsequent research.

In conclusion, the close collaboration between journals and the high citation rates of scholars reflect the widespread influence and rapid development of the heart sensor field within the global academic community. Furthermore, cross-journal and interdisciplinary cooperation will further promote innovation and technological breakthroughs in this field, providing more opportunities for the future optimization of heart health monitoring technologies.

### 3.7 Dataset of harnessing AIoT for cardiac sensing

The development and rigorous validation of AIoT-based cardiac sensing solutions critically depend on publicly available datasets that capture a variety of physiological modalities, acquisition conditions, and subject cohorts. As summarized in Table 1, five benchmark datasets have become de facto standards for algorithm development and comparative evaluation in this field. These repositories span PPG, ECG, multimodal recordings, and motion artefact labels, thereby supporting a wide range of use cases from heart-rate estimation under motion to arrhythmia detection in clinical-grade signals.

**Table 1:** Available dataset of cardiac sensing.

Dataset	Description	Cardiac sensing data included
PPG-DaLiA (63)	PPG signals from wrist-worn devices during daily activities (e.g., sitting, walking, cycling). Includes motion data for context-aware analysis. URL: https://ubicomp.eti.unisiegen.de/home/datasets/sensors19/	Fifteen subjects performed a range of activities under near-real-life conditions
TROIKA Dataset (64)	Wrist-based PPG and accelerometer data for heart rate estimation during physical exercise. URL: https://arxiv.org/abs/1409.5181	12 subjects during fast running at the peak speed of 15 km/h
WESAD (Wearable Stress and Affect Detection) (65)	Multimodal data (ECG, PPG, EDA) from chest (RespiBAN) and wrist (Empatica E4) devices. Focuses on stress and emotion detection. URL: https://archive.ics.uci.edu/dataset/465/wesad+wearable+stress+and+affect+detection	Instances: 63000000
CapnoBase Dataset (66)	Simultaneous PPG and ECG recordings from pulse oximeters and clinical devices. URL: https://peterhcharlton.github.io/RRest/datasets.html	39 subjects’ data analysed
MIT-BIH Arrhythmia Database (67)	Classic ECG dataset from Holter monitors (portable devices). URL: https://www.physionet.org/content/mitdb/1.0.0/	Sample: 590262

The PPG-DaLiA dataset comprises wrist-worn PPG and tri-axial accelerometer recordings from fifteen healthy volunteers during daily activities (e.g., sitting, walking, cycling), offering context-aware labels for motion-robust heart-rate estimation. In contrast, the TROIKA dataset focuses on high-intensity exercise, providing PPG and accelerometry from twelve subjects running at speeds up to 15 km/h, which is ideal for benchmarking algorithms under pronounced motion artefacts. The WESAD (Wearable Stress and Affect Detection) dataset extends beyond cardiac sensing to include chest-mounted ECG and PPG, wrist-worn PPG and electrodermal activity (EDA), and skin temperature measurements across stress, amusement, and baseline states (≈63 million instances), facilitating multimodal emotion and stress detection studies. The CapnoBase dataset offers synchronized PPG and clinical-grade ECG recordings from 39 subjects, enabling cross-modal fusion and quality-assessment research. Finally, the classic MIT-BIH Arrhythmia Database, with over 590,000 annotated ECG beats from Holter monitors, remains the gold standard for arrhythmia classification.

Collectively, these datasets underpin AIoT cardiac sensing research by providing diverse signal types, rich annotations, and real-world contexts. Their complementary characteristics—from daily-life motion labels to high-fidelity clinical ECG—ensure that AIoT algorithms can be trained, validated, and stress-tested across the full spectrum of application scenarios.

### 3.8 Machine learning for harnessing AIoT for cardiac sensing

Machine learning (ML) techniques play a central role in transforming raw physiological signals acquired via AIoT platforms into clinically relevant insights. As summarized in Table 2, both classical and deep learning algorithms have been applied to tasks ranging from disease prediction and arrhythmia classification to continuous anomaly detection under real-world conditions.

**Table 2:** Summary of machine learning for cardiac sensing.

Reference	Main ideas	Applied method	Performance
Singh and Kumar (25)	Examines several popular machine learning models for heart disease prediction.	Logistic Regression, KNN	88.5% accuracy
Stehlik et al. (27)	Analysed data from a wearable patch for predicting the risk of rehospitalization.	smartphone-based and cloud-based machine learning algorithm	88% sensitivity
De Cannière et al. (28)	Multi-parameter sensors (ECG and accelerometer) were used to collect data during each test.	Linear Regression, Support Vector Machine	Linear Model: R^2^ of 0.661, RMSE of 64.8 m, Best performing model had a MAE of 42.8 m (±36.8 m)
Feng et al. (29)	Piezoelectric sensor captured BCG and respiratory effort signals; linear/nonlinear features extracted for HF detection; improved performance validated via LOO/LOSO cross-validation with ML classifiers.	KNN, SVM, RF (Random Forest), XGBoost	accuracy of 94.97 and 87.00% in the LOO and the LOSO experiments
Nigar et al. (30)	proposes a hybrid approach that combines the Internet of Medical Things (IoMT) and ML for the early detection and monitoring of six chronic diseases	VGG16, VGG19, ResNet, DenseNet and Inception-v3	88.3% AUC for Heart Disease
Renu et al. (26)	Prediction of heart disease using various machine learning algorithms.	K-NN, Decision Trees, SVM, Logistic Regression, Random Forest	the KNN has the greatestaccuracy of 100%
Vani (68)	Impact of machine learning in cardiac disease diagnosis.	SVM, Naive Bayes	
Yadav et al. (69)	Prediction of cardiac arrest using machine learning.	Univariate and Bivariate analysis	
Ekuma et al. (70)	Classification of cardiac disease using ML.	Logistic Regression, K-NN, SVM, Naive Bayes, Decision Tree, Gradient Boosting	High accuracy with confusion matrix evaluation
Kwon and Dong (38)	flexible sensors and ML for heart monitoring.	Flexible cardiac sensors with machine learning	Promising results for real-time cardiac monitoring
Sarveshvar et al. (71)	Comparison of different ML techniques for heart disease prediction.	Naïve Bayes, Logistic Regression, Random Forest	Promising outcomes with accuracy and confusion matrix validation
Mayourian et al. (33)	AI-ECG model to predict cardiovascular magnetic resonance (CMR)-defined biventricular dysfunction/dilation in patients with CHD.	convolutional neural network, CNN	AUROC: LV dysfunction 0.89; LV dilation 0.83; RV dysfunction 0.82; RV dilation 0.80
Mayourian et al. (34)	The model used a CNN trained on ECG–echocardiogram pairs from patients ≤18 years of age, collected within 2 days apart.	convolutional neural network, CNN	LV Composite Outcome: AUROC 0.86, AUPRC 0.39 LV Dysfunction: AUROC 0.94, AUPRC 0.32 LV Hypertrophy: AUROC 0.84, AUPRC 0.25, LV Dilation: AUROC 0.87, AUPRC 0.33
Siontis et al. (72)	The purpose of this study was to determine the frequency, associations, and prognostic impact of different clinical presentations of new-onset AF.	Mod-Sev MR, confirmed by echo	AUC: IV 0.758, EV 0.850
Attia et al. (32)	AI-enabled ECG screening method for identifying asymptomatic left ventricular dysfunction (ALVD).	Convolutional Neural Network	AUC: HCM-0.91, PAH-0.94, Amyloid-0.86, MVP-0.77
Su et al. (31)	a portable ECG signal acquisition and analysis system based on machine learning and model fusion.	logistic regression, support vector machines, XGBoost, CNN, LSTM	overall classification accuracy of 99.13%
Cañón-Clavijo et al. (73)	IoT-based system for heart monitoring and arrhythmia detection.	CNN, k-nearest neighbors (KNN), and RF	Normal Beats: 93% Ventricular Beats: 94% Supraventricular Beats: 82%
Rincon et al. (36)	IoT and fog computing-based monitoring system for cardiovascular patients.	Deep Learning, MobileNet	Atrial Fibrillation (Af): 90%, Normal Sinus Rhythm (Nsr): 89%, Too Noisy to Classify (no): 92%, Other Rhythm (Or): 95%
Sivapalan et al. (37)	Model is designed to address several key challenges in real-time ECG anomaly detection for IoT edge devices.	LSTM, MLP	94% Accuracy, 85% F1 score
Wong et al. (35)	Efficient binary convolutional neural network (bCNN) algorithm utilizing function-merging and block-reuse techniques to classify between Ventricular and non-Ventricular Ectopic Beat images.	bCNN	97.3% accuracy, 91.3% sensitivity, 98.1% specificity, 86.7% precision, 88.9% F1-score

Early studies focused on traditional classifiers for binary heart-disease prediction and risk stratification. For example, Singh and Kumar (25) compared logistic regression and k-nearest neighbors (KNN), achieving up to 88.5% accuracy in predicting coronary artery disease, while Renu et al. (26) reported 100% accuracy for KNN on a similar task. Stehlik et al. (27) employed smartphone-based and cloud-based ML pipelines to predict rehospitalization risk in heart-failure patients, obtaining 88% sensitivity. De Cannière et al. (28) integrated ECG and accelerometer data with support vector machines (SVM) and linear regression models, yielding an R^2^ of 0.661 and root-mean-square error (RMSE) of 64.8 m for rehabilitation tracking. These conventional methods offer interpretability and low computational overhead, making them suitable for resource-constrained AIoT devices.

Beyond classical approaches, ensemble and tree-based models such as random forests (RF) and XGBoost have been applied to biosignal features for heart-failure detection. Feng et al. (29) extracted linear and nonlinear features from ballistocardiography and respiratory effort signals, demonstrating up to 94.97% accuracy in leave-one-out experiments with RF and XGBoost classifiers. Similarly, Nigar et al. (30) proposed a hybrid IoMT–ML framework combining convolutional neural network (CNN) backbones (e.g., VGG16, ResNet) with edge-to-cloud analytics, achieving an area under the ROC curve (AUC) of 0.883 for heart-disease detection.

Deep learning architectures have further advanced AIoT cardiac sensing by enabling end-to-end waveform analysis. Su et al. (31) developed a portable ECG acquisition and model-fusion system incorporating CNN and long short-term memory (LSTM) networks, reporting 99.13% overall classification accuracy. Attia et al. (32) employed a CNN to screen for asymptomatic left ventricular dysfunction, obtaining AUCs up to 0.94 across multiple cardiomyopathies. Recent pediatric studies by Mayourian et al. (33, 34) leveraged CNNs on ECG–echocardiogram pairs to predict biventricular dysfunction, achieving AUCs between 0.80 and 0.94 for various outcomes. Wong et al. (35) introduced a binarized CNN (bCNN) for ventricular ectopic beat classification on edge devices, recording 97.3% accuracy and 98.1% specificity.

IoT-specific implementations have also been explored. Rincon et al. (36) integrated a MobileNet-based deep network with fog computing for real-time ECG classification, achieving over 90% accuracy across rhythm classes. Sivapalan et al. (37) demonstrated a lightweight LSTM–MLP anomaly detector optimized for IoT edge sensors, yielding 94% accuracy and 85% F1-score in live deployments. Kwon and Dong (38) combined flexible piezoelectric sensors with ML algorithms to enable continuous, non-invasive cardiac monitoring.

Collectively, these studies illustrate the breadth of ML strategies—from interpretable linear models to compact deep neural networks—deployed on AIoT infrastructures for cardiac sensing. While classical methods remain valuable for low-power applications, deep learning offers superior performance for complex waveform analysis. Future research should address challenges in data heterogeneity, on-device inference efficiency, and model explainability to facilitate robust, real-time cardiac monitoring across diverse populations.

## 4 Discussion

Our analysis demonstrates a 500% increase in AIoT–cardiac sensing publications from 2018 to 2024, with ‘machine learning,’ ‘deep learning,’ and ‘wearable sensors’ emerging as the most central keywords. These trends underscore the rapidly growing integration of AI algorithms into IoT-based cardiac monitoring platforms (Figures 2a,e) (39, 40). It illuminates current advancements, collaboration networks, and key thematic clusters. The surge in scientific output over the past five years reflects growing scholarly and industrial interest in integrating advanced artificial intelligence techniques with IoT-based healthcare solutions for cardiovascular disease.

A notable finding is that the United States and China have emerged as leaders in both research productivity and recognition, a trend that aligns with substantial national-level investments in AI research and medical innovation (18, 41). While these two countries demonstrate robust research output, the majority of collaborative activities remain concentrated within their respective national networks, highlighting a missed opportunity for deeper global partnerships (42–44). In contrast, European countries/regions, Japan, and the Taiwan Province of China have engaged in more frequent cross-border collaborations, suggesting that broader international efforts could accelerate technological breakthroughs and promote standardized methodologies in this field.

Another critical insight into current advancements in this field is the central role of deep learning (45), machine learning, and neural network methods in processing large-scale cardiac data. Techniques such as CNNs, LSTM networks, and other advanced algorithms have revolutionized the detection of cardiac anomalies, including arrhythmias, and have improved predictive capabilities through the analysis of ECG signals and heart rate variability. Achieving reliable performance in real-world settings depends heavily on robust sensor technologies and consistently high-quality data. Future efforts may benefit from refining the design of wearable and implantable sensors while advancing data collection protocols to mitigate potential variability and ensure reproducible model training.

The integration of IoT devices with AI-driven analysis has transformed traditional approaches to cardiovascular diagnostics and disease management. Real-time physiological monitoring and wireless data transmission enable more efficient care models, supporting remote patient monitoring, early intervention, and personalized therapy recommendations. Edge computing techniques (10, 46), in particular, hold significant promise for reducing latency, improving computational efficiency, and addressing privacy concerns. Scaling these technologies will require a robust approach to infrastructure development, including secure data-sharing protocols, cloud or edge computing architectures, and regulatory frameworks to ensure patient privacy and data security (47), particularly in the era of large language models (LLMs) (48). For instance, Abbott’s CardioMEMS HF System—a wireless implantable pulmonary artery pressure sensor—has been deployed in home-care programs, reducing heart failure readmissions by up to 37% (13). In contrast to RAG-based pipelines such as ClinicalRAG (49), which augments LLM outputs with dynamically retrieved heterogeneous medical knowledge to improve diagnostic accuracy, and MedRAG (50), which leverages knowledge-graph–elicited reasoning to reduce chronic-pain misdiagnosis by up to 12%, our edge-enabled IoT–AI framework uniquely integrates real-time physiological sensor streams with low-latency, on-device analytics.

Despite encouraging progress, several challenges remain. Chief among these are ensuring patient data security and achieving model interpretability in high-stakes clinical settings. The transfer and storage of sensitive health information carry inherent risks, highlighting the need for robust cryptographic mechanisms and carefully designed privacy protocols (9). Additionally, the “black-box” nature of many AI models raises concerns for clinical adoption (51), as healthcare providers and regulatory bodies increasingly demand transparent and explainable decision-making processes (52). Advances in interpretable or explainable AI could address these concerns by offering greater clarity into how AI-based decisions are made, thereby fostering trust among clinicians and patients. For example, embedding SHAP-based explanations in edge-deployed wearable ECG analytics can highlight the specific waveform features driving arrhythmia predictions, allowing clinicians to interpret and validate model outputs in real time (53). Future work should explore AIoT integration into home-based cardiac kits and telehealth platforms—such as Eko’s DUO digital stethoscope with 1-lead ECG for remote cardiac exams and the PrediHealth telemonitoring kit combining medical and environmental sensors with AI-driven predictive models for chronic heart failure management (54). This perspective article also has certain limitations. Although the reliance on the Web of Science database is well-justified given its reputation, it may exclude significant scientific contributions published in conference proceedings (55), smaller specialized journals, or patent literature within this field.

In summary, current advancements in AI-powered IoT cardiac sensing center on refining AI algorithms—notably deep learning architectures—and advancing the capabilities of smart wearable devices to support precision medicine (56–61). In our assessment, the field must now shift toward prioritizing data security and privacy safeguards, especially as LLMs introduce new ethical and technical complexities (48). Addressing these challenges will be critical to ensuring sustainable progress and societal trust. Moving forward, integrating AIoT-driven cardiac sensing into public health frameworks remains essential to facilitate early detection of cardiovascular diseases, ultimately improving global healthcare outcomes through innovation, collaboration, and responsible governance. Moreover, AIoT-driven solutions such as handheld tele-ECG systems and cloud-connected wireless Holter monitors promise to bridge cardiac care gaps in rural and resource-limited settings by enabling real-time diagnosis and remote specialist consultation (62).

## Data Availability

The data presented in this study are deposited in the GitHub repository “Drcreater/review” (https://github.com/Drcreater/review).
